# Variation in lumbar punctures for early onset neonatal sepsis: a nationally representative serial cross-sectional analysis, 2003-2009

**DOI:** 10.1186/1471-2431-12-134

**Published:** 2012-08-28

**Authors:** Stephen W Patrick, Robert E Schumacher, Matthew M Davis

**Affiliations:** 1 Department of Pediatrics and Communicable Diseases, University of Michigan Health System, Ann Arbor, Michigan, 48109, USA; 2 Robert Wood Johnson Foundation Clinical Scholars Program, University of Michigan Health System, Ann Arbor, Michigan, 48109, USA; 3 Division of Neonatal-Perinatal Medicine, University of Michigan Health System, Ann Arbor, Michigan, 48109, USA; 4 Child Health Evaluation and Research (CHEAR) Unit, Department of Pediatrics and Communicable Diseases, University of Michigan Health System, Ann Arbor, Michigan, 48109, USA; 5 Gerald R. Ford School of Public Policy, University of Michigan, Ann Arbor, Michigan, 48109, USA; 6 6312 Medical Science Building 1 1150 W. Medical Center Drive SPC 5604, Ann Arbor, Michigan, 48109-5604, USA

**Keywords:** Neonatal, Sepsis, Lumbar puncture, Variation, Early onset neonatal sepsis

## Abstract

**Background:**

Whether lumbar punctures (LPs) should be performed routinely for term newborns suspected of having early onset neonatal sepsis (EONS) is subject to debate. It is unclear whether variations in performance of LPs for EONS may be associated with patient, hospital, insurance or regional factors. Our objective was to identify characteristics associated with the practice of performing LPs for suspected EONS in a nationally representative sample.

**Methods:**

Utilizing data from the 2003, 2006 and 2009 Kids’ Inpatient Database (KID) compiled by the Agency for Healthcare Research and Quality, we examined the frequency and characteristics of term, normal-birth weight newborns receiving an LP for EONS. Survey-weighting was applied for national estimates and used in chi squared and multivariable regression analysis.

**Results:**

In 2009, there were 13,694 discharges for term newborns that underwent LPs for apparent EONS. Newborns having LPs performed were more likely to be covered by Medicaid vs. private insurance (51.9 vs. 45.1 percent; p < 0.001), be born in urban vs. rural hospitals (94.8 vs. 87.3 percent; p < 0.001), teaching vs. non-teaching (60.8 vs. 43.1 percent; p < 0.001) and children’s hospitals vs. non-children’s (23.0 vs. 11.2 percent; p < 0.001). Lastly, newborns having LPs performed were disproportionately born in the Northeast census region (p = 0.03). In multi-year adjusted analysis, infants with Medicaid coverage, and those born in urban or teaching hospitals, consistently had higher odds of having an LP performed.

**Conclusions:**

We found pronounced variation in LPs performed for EONS, even when adjusting for clinical conditions that would prompt LPs. These findings indicate practice variations in newborn care that merit further examination and explanation.

## Background

Lumbar punctures (LPs) are commonly performed on newborns suspected of having sepsis [1]. Over the last thirty years, numerous studies have evaluated the utility of LPs in identifying early onset neonatal sepsis (EONS). While several studies found that LPs were unnecessary among asymptomatic newborns suspected of having EONS for risk factors related to maternal reasons [2-4] or with respiratory signs [5,6], other analyses found that cases of meningitis were missed when an LP was not performed on this population [7,8].

In 2002, the American Academy of Pediatrics (AAP) and the Centers for Disease Control and Prevention (CDC) [9] released guidelines for the diagnostic approach of a newborn suspected of having EONS attributable to Group B *Streptococcus* (GBS), the most common causative organism among normal-birth weight infants [1,10]. These guidelines state that LPs should be reserved for newborns with “signs” of a systemic infection and should not be performed on newborns without “signs”. Despite this, “signs” of a systemic infection can be subtle such as poor feeding, irritability or a high-pitched cry, leading to some variability in the diagnosis [1,11]. In addition, it is unclear how broadly these guidelines have been adopted.

Institutional variation in practice has previously been described in newborn care [12,13]. Given conflicting data about the utility of performing an LP for EONS, we hypothesize that there is variation in LPs among institutions caring for newborns related to factors such as teaching status or to regional location. Because normal-birth weight infants are less likely than low-birth weight infants to be born with co-morbid conditions that might prompt an LP, we sought to evaluate LP variation among only normal-birth weight newborns. To address these issues in a broad sample, we utilized multiple years of a nationally representative dataset to assess whether the practice of performing LP for EONS varied by child, hospital or insurance characteristics for normal-birth weight infants.

## Methods

### Data source

Data were obtained from the Agency for Healthcare Research and Quality’s (AHRQ) Kids’ Inpatient Database (KID) from 2003, 2006 and 2009 (the most recent year available). The KID is part of a broad set of databases developed by AHRQ as part of a federal-state-industry partnership through the Healthcare Cost and Utilization Project (HCUP). HCUP includes the largest collection of hospital care data in the United States and includes all payers.

KID was specifically designed to evaluate a broad range of conditions and procedures affecting children. It is the only national hospital administrative dataset specifically designed to assess use of hospital services by newborns. KID is compiled in order to obtain a nationally representative sample, which allows for sufficient statistical power to evaluate rare conditions and procedures. This sample includes 80 percent of pediatric discharges and 10 percent of all uncomplicated births [14].

### Subjects

Utilizing the KID, we examined the frequency and the characteristics of newborns that underwent an LP for EONS. Given that nearly all LPs for newborns are performed within the first week of life and that treatment of meningitis is generally 14 to 21 days [15], we limited our sample to newborns whose length of stay was less than 21 days. Newborns weighing less than 2500 grams or who were transferred from another facility were excluded from the analysis.

Using the *International Classification of Diseases, Ninth Revision, Clinical Modification* (ICD-9 CM), we identified neonatal diagnoses and procedures. To first identify our sample, infants with LPs performed were identified using ICD-9 procedure code (03.31). Clinical characteristics of the sample of newborns were then identified using ICD-9 CM codes: any infection (771.0–771.8), temperature instability (778.0–778.9), meningitis (320.0–320.9, 321.0–321.8, 322.0–322.8, 036.0) and respiratory diagnoses (769, 770.0–770.9). Rates of LPs performed and meningitis diagnoses are expressed as per 100,000 hospital births.

Newborn characteristics were further categorized as male or female. Race/ethnicity was missing for more than 25% of newborns and was therefore excluded from the analysis. Insurance was categorized as private insurance, Medicaid, self-pay (uninsured) and other (Tricare, etc.). Utilizing the newborn’s home zip code, median income for that zip code was provided in the KID and further subdivided into national quartiles. Birth weight was determined through diagnostic data provided in the dataset, described elsewhere [15].

### Hospital characteristics

Hospital characteristics were assigned using variables provided in the KID dataset. Using the National Association of Children’s Hospitals and Related Institutions (NACHRI) classification, hospitals were defined as 1) not a children’s hospital, 2) children’s general hospital, 3) children’s specialty hospital, 4) children’s unit in a general hospital. For our analysis, we defined “children’s hospital” as groups 2 or 4. Hospitals were further defined as urban or rural (less than 500 people per square mile), based upon United States Census Bureau data, and teaching or non-teaching based upon the American Hospital Association Annual Survey Database.

### Data analyses

The authors conducted all analyses using Stata 12.0 (Stata Corp, College Station, TX) statistical software. For all analyses, we applied sampling weights provided by the KID [14] to generate nationally representative estimates. The complex survey design accounts for clustering of data at the hospital level. All results are reported utilizing weighted estimates in each study year. Under the regulations of the University of Michigan Medical Institutional Review Board, as a study of de-identified data this study was exempt from human subjects review.

Survey-weighted logistic regression was performed to assess hospital characteristics associated with LP for EONS, before and after controlling for newborn clinical characteristics and newborn demographics. This analysis was repeated to evaluate payer and hospital region associated with LP for EONS. Records with missing data were excluded from multivariate analyses.

### Sensitivity analysis

All analyses were repeated separately to include newborns whose length of stay was less than 7 days and 28 days (rather than 21 days as described above). Our results for both analyses were similar to the results we report below, and are therefore not reported separately; they are available from the corresponding author upon request.

## Results

### Sample characteristics

In 2009, there were an estimated 13,694 LPs performed for EONS out of a total of nearly 3.7 million term newborns. Newborns having LPs performed were less likely to be female (43.1 vs. 48.6 percent; p < 0.001), more likely to have a respiratory diagnosis (50.8 vs. 5.9 percent; p < 0.001), any infection (49.2 vs. 1.3 percent; p < 0.001), temperature instability (14.0 vs. 3.3 percent; p < 0.001) and have a diagnosis of meningitis (2.7 vs. <0.01 percent; p < 0.001). In addition, those undergoing LPs were more likely to be covered by Medicaid vs. private insurance (51.9 vs. 45.1 percent; p < 0.001). Additionally, several hospital differences were noted; LPs were more common in urban (94.8 vs. 87.3 percent; p < 0.001), teaching (60.8 vs. 43.1 percent; p < 0.001) and children’s hospitals (23.0 vs. 11.2 percent; p < 0.001). Lastly, newborns having LPs performed were disproportionately born in the Northeast census region (p = 0.03) (Table 1).

**Table 1:** Characteristics of newborns with lumbar punctures being performed versus all other newborns in the United States, 2009

***Newborn Characteristics***		
	**Term newborns with LP 13,694**	**All other term newborns 3,684,437**
**National Estimated Total**	**Percent (95% CI)**	**Percent (95% CI)**
**Gender**		
Female	43.1 (42.0–44.2)	48.6 (48.5–48.8)
**Payer type**		
Medicaid	51.9 (49.4–54.4)	45.1 (43.8–46.4)
Private	41.3 (38.6–44.1)	47.8 (46.4–49.1)
Uninsured	3.9 (3.1–4.8)	4.4 (4.0–4.8)
Other	3.0 (2.3–3.9)	2.8 (2.5–3.0)
**Income Quartile**		
First (<40 k, US$)	30.9 (27.7–34.2)	27.8 (26.5–29.0)
Second (40–49 k, US$)	24.2 (22.0–26.5)	26.2 (25.4–27.1)
Third (50–65 k, US$)	24.0 (22.4–25.7)	24.9 (24.1–25.7)
Fourth (>66 k, US$)	21.0 (17.2–25.4)	21.1 (19.9–22.5)
**Hospital type**		
Urban	94.8 (93.6–95.7)	87.3 (86.6–87.9)
Teaching	60.8 (56.0–65.4)	43.1 (41.3–44.9)
Children’s	23.0 (17.0–30.3)	11.2 (9.2–13.6)
**Hospital Region**		
South	38.5 (33.9–49.4)	38.4 (36.7–40.1)
Northeast	21.2 (17.8–25.1)	15.9 (14.8–17.0)
West	20.9 (16.0–26.8)	24.2 (22.9–25.4)
Midwest	19.4 (16.2–23.0)	21.6 (20.5–22.7)
**Diagnosis**		
Any respiratory diagnosis	50.8 (49.0–52.5)	5.9 (5.8–6.1)
Any infection	49.2 (46.4–51.9)	1.3 (1.2–1.4)
Temperature instability	14.0 (12.7–15.3)	3.3 (3.1–3.5)
Meningitis	2.7 (2.2–3.3)	0.0017 (0.0012–0.0023)

### Rates of lumbar puncture and meningitis among normal-birth weight neonates

In 2009, there was variation in the number of LPs for apparent EONS and subsequent diagnosis of meningitis by insurance and hospital type. Among payers, Medicaid had the highest rate of LPs (426.3 per 100,000 Medicaid births; 95%CI 394.9–455.2) and meningitis (14.0 per 100,000 Medicaid births; 95%CI 11.5–16.4), compared to private insurance with the lowest LPs (320.7 per 100,000 privately insured births; 95%CI 293.4–345.6) and meningitis rate (9.1 per 100,000 privately insured births; 95%CI 7.4–10.7). Urban, teaching and children’s hospitals had higher rates of LPs and meningitis. Lastly, infants born in the Northeast had the highest rate of LPs performed (493.6 per 100,000 Northeast births; 95%CI 434.1–545.1) compared to the West with the lowest (320.3 per 100,000 West births; 95%CI 233.8–397.5). Notably, there was no statistical difference in the diagnosis of meningitis between census regions (Table 2).

**Table 2:** Rates of LPs performed for presumed EONS and meningitis diagnoses per 100,000 term newborns, 2009

	**LP rate (95% CI)**	**Meningitis rate (95% CI)**
**Payer type**		
Medicaid	426.3 (394.9–455.2)	14.0 (11.5–16.4)
Private	320.7 (293.4–345.6)	9.1 (7.4–10.7)
Uninsured	328.4 (283.4–365.9)	8.4 (1.7–14.0)
Other	395.2 (314.9–462.0)	23.5 (–2.6–45.2)
**Hospital type**		
Rural	151.5 (128.8–172.2)	6.1 (3.1–8.8)
Urban	399.5 (369.2–427.7)	12.4 (10.0–14.7)
Non-teaching	254.0 (231.0–275.4)	8.0 (6.5–9.5)
Teaching	518.1 (466.9–563.3)	16.4 (11.8–20.4)
Non-children’s	325.0 (303.0–345.3)	10.7 (8.5–12.8)
Children’s	769.2 (626.3–863.3)	19.9 (10.0–26.4)
**Hospital region**		
South	371.5 (336.5–402.5)	11.8 (8.9–14.3)
Northeast	493.6 (434.1–545.1)	10.6 (7.6–13.1)
West	320.3 (233.8–397.5)	14.0 (7.2–20.1)
Midwest	333.1 (287.3–374.2)	9.7 (5.7–13.3)

### Trends in adjusted odds of lumbar puncture for EONS, 2003–2009

After adjusting for potential confounding variables such as respiratory diagnoses, temperature instability, any infection and median income of newborn’s zip code, newborns born in urban areas and teaching hospitals were significantly more likely to have LPs for EONS than comparison newborns in all study years. In 2003, there was a trend towards children’s hospitals performing more LPs than non-children’s hospitals; in 2006 and 2009 this finding persisted and was statistically significant. In addition, infants enrolled in state Medicaid programs were more likely to have an LP performed than those who were privately insured in all study years. In 2006, uninsured infants were also more likely to have an LP than those who were privately insured. Lastly, infants born in the Northeast were more likely than those in the West to have LPs in 2003 and 2006, and there was a trend to more LPs in the Northeast in 2009 (Table 3).

**Table 3:** Adjusted and unadjusted odds of lumbar puncture for EONS, by payer, hospital type and region, 2003–2009

	**2003**		**2006**		**2009**	
	**Unadjusted OR (95% CI)**	Adjusted* OR (95% CI)	**Unadjusted OR (95% CI)**	Adjusted* OR (95% CI)	**Unadjusted OR (95% CI)**	Adjusted* OR (95% CI)
**Payer type**						
Private insurance	ref	ref	ref	ref	ref	ref
Medicaid	1.3 (1.1–1.5)	1.2 (1.1–1.4)	1.4 (1.2–1.6)	1.4 (1.2–1.5)	1.3 (1.2–1.5)	1.2 (1.1–1.4)
Uninsured	1.2 (0.9–1.7)	1.3 (0.9–1.9)	1.3 (1.0–1.6)	1.4 (1.1–1.7)	1.0 (0.8–1.3)	1.0 (0.8–1.3)
Other	2.2 (1.1–4.3)	2.2 (1.1–4.1)	1.1 (0.9–1.3)	1.1 (0.9–1.3)	1.2 (0.9–1.6)	1.1 (0.9–1.5)
**Hospital type**						
Rural	ref	ref	ref	ref	ref	ref
Urban	2.4 (1.9–3.0)	1.6 (1.3–2.0)	2.7 (2.2–3.3)	1.7 (1.3–2.1)	2.6 (2.2–3.2)	1.8 (1.4–2.3)
Non-teaching	ref	ref	ref	ref	ref	ref
Teaching	2.1 (1.8–2.6)	1.5 (1.2–2.0)	2.2 (1.9–2.6)	1.7 (1.4–2.0)	2.0 (1.7–2.5)	1.6 (1.4–2.0)
Non-children’s Hospital	ref	ref	ref	ref	ref	ref
Children’s Hospital	2.1 (1.6–2.9)	1.4 (0.95–2.0)	2.4 (1.8–3.3)	1.6 (1.1–2.3)	2.4 (1.7–3.3)	1.6 (1.1–2.3)
**Hospital region**						
West	ref	ref	ref	ref	ref	ref
Midwest	1.3 (1.0–1.8)	1.4 (1.0–1.8)	1.3 (.95–1.8)	1.3 (0.9–1.9)	1.0 (0.7–1.5)	1.1 (0.8–1.6)
South	1.4 (1.1–1.9)	1.2 (0.9–1.7)	1.3 (1.0–1.8)	1.2 (0.9–1.7)	1.2 (0.8–1.6)	1.1 (0.8–1.5)
Northeast	2.2 (1.5–3.1)	1.9 (1.3–2.8)	2.0 (1.3–2.9)	1.7 (1.1–2.6)	1.5 (1.1–2.2)	1.4 (0.9–2.1)

* adjusted for payer, hospital type, hospital region, income quartile of the newborn, respiratory diagnosis, any infection and temperature instability.

Ref = reference category.

## Discussion

Our analysis is the first to describe national variation in performance of LPs for EONS among normal-birth weight infants. These variations are significant and persist even with adjustments for potential confounding factors. Such variations in care indicate inconsistent application of available clinical guidelines, and also suggest broad opportunities for improvement in the delivery of neonatal care related to concerns about infection.

Lumbar punctures are invasive procedures and have been noted to be more difficult to perform in neonates than in other populations and to cause neonatal distress [16]. In this population, complications such as “bloody taps” [17] and contaminated specimens [5,6] are common, and therefore limiting a newborn’s exposure to an LP may be desirable. On the other hand, some studies suggest that LPs performed only on newborns with signs of infection lead to missed diagnoses of meningitis and its associated morbidity [7,8]. Still other studies have found discordance of cerebral spinal fluid and blood cultures in septic patients [18], suggesting all newborns suspected of EONS should have LPs.

Both the CDC [9,19] and the AAP [15] have published guidelines to assist clinicians in the diagnostic evaluation of a newborn suspected of having GBS EONS. Variation in LPs in this analysis suggests that adoption of these guidelines is uneven. While it is impossible to delineate from our findings which group is performing the “clinically appropriate” number of LPs, inconsistency across clinicians practicing in different settings is evident.

Variation in both practice [12,13] and clinical outcomes [20,21] among neonatal intensive care units has been described previously. However, no study has exclusively compared children’s hospitals versus non-children’s hospitals when comparing LPs performed on neonates. Our study finds that LPs were done more frequently in children’s hospitals in two of our three study years, even after controlling for confounders. Even with this, it is possible that differences between children’s and non-children’s hospitals found in our study occurred because of unexplained confounding. For example, despite our broad inclusion of clinical diagnoses, it is plausible that newborns admitted to children’s hospitals have more serious or more complex illness, prompting more frequent LPs.

Our study also found some patient-level variables associated with LPs for EONS – in particular, insurance status. In one study year (2006), uninsured newborns were more likely to have LPs performed than those who were privately insured. This is potentially linked to maternal insurance status in the antenatal period. Mothers who are uninsured are less likely to utilize prenatal services [22], thus less is known about their risks of peripartum infection. In this setting of uncertainty, neonatal providers might be more likely to perform LPs. We also found that newborns with Medicaid were more likely to have an LP for EONS in all study years. In this case, it is possible that insurance status is a proxy for race/ethnicity, because national enrollment data indicate that black and Hispanic children have proportionately higher rates of enrollment in Medicaid [23]. It has been previously described that minority groups are disproportionately affected by GBS EONS [10,19], therefore leading to the possibility that they were more likely to undergo LPs to evaluate for the condition.

Our study found geographic variation in the practice of performing LPs for EONS, with the Northeast having a statistically significant higher rate of LPs performed in 2003 and 2006, and a trend to more LPs in 2009. While no prior study has evaluated geographic variation in the practice of LPs for EONS, previous analyses have found geographic differences in other procedures such as placement of intracranial pressure monitors [24] and tracheostomy [25]. Such regional variation may reflect variation in pediatrician and/or neonatologist training or differences in epidemiologic patterns that influence clinicians’ perception of EONS risk.

Our study finds that more LPs sometimes led to more diagnoses of meningitis. As an example, newborns enrolled in state Medicaid programs were more likely than privately insured infants to have an LP performed and were more likely to have a diagnosis of meningitis. This finding also occurred in urban and teaching hospitals. In contrast, our study found that LPs were more commonly performed in the Northeast with no difference noted in the diagnosis of meningitis among the four census regions. One would expect that more LPs would lead to more diagnoses of meningitis; however, this finding was not consistent, suggesting the possibility that excess LPs are being performed. This inconsistency merits further study.

In fall of 2010, the CDC released updated guidelines for the diagnostic evaluation of newborns at risk of GBS EONS. The guidelines clarify that only a limited evaluation (blood culture and empiric antibiotics) should be completed for a newborn without signs of sepsis whose mother is diagnosed with chorioamnionitis (no LP). Additionally, it recommends observation for term newborns who are well-appearing without prolonged rupture of membranes (even if their mothers met criteria for intrapartum antibiotics and they were not received), potentially limiting antibiotic use in this group [19]. The introduction of these new recommendations and the clarifications in the treatment algorithm provided in the guidelines might lead to more standard care and ensure LPs are performed judiciously.

### Limitations

There are limitations to our study common to analyses of secondary data. Our study utilized a nationally representative administrative dataset, which relies on hospital discharge abstracts. The use of these data allows for nationally representative inferences, but administrative data are subject to errors of omission and commission related to coding discrepancies. We did not have access to medical records for these patients that would have allowed us to validate the codes. Because race was missing in approximately one-quarter of our data, we were not able to evaluate the potential impact of race on our primary outcome, an important limitation.

In addition, discharge abstracts do not contain admitting diagnoses. Final billing diagnoses may not fully reflect clinical conditions or co morbidities present at the time of birth that may have influenced clinical decisions such as LPs. On the other hand, discharge abstracts likely reflect conditions most relevant to the clinical course of the infants. KID data are available at the encounter level rather than the patient level; therefore, we are unable to evaluate readmissions to look for possible missed cases of meningitis.

## Conclusion

Caring for a neonate suspected of having EONS can be a diagnostic dilemma for clinicians, given that presenting signs can be largely subjective. In this national analysis, we found pronounced institutional variation in LPs performed for EONS, even when adjusting for hospital differences and clinical conditions that would prompt LPs. Our findings prompt calls for further standardization, limiting unnecessary procedures for newborns.

## Abbreviations

(LPs): Lumbar punctures; (EONS): Early onset neonatal sepsis; (AAP): American Academy of Pediatrics; (CDC): Centers for Disease Control and Prevention; (GBS): Group B *Streptococcus*; (AHRQ): Agency for Healthcare Research and Quality’s; (KID): Kids’ Inpatient Database; (HCUP): Healthcare Cost and Utilization Project; (NACHRI): National Association of Children’s Hospitals and Related Institutions.

## Competing interests

The authors declare that they have no competing interests.

## Authors’ contribution

All authors (SP, MD, RS) contributed to the analysis, interpretations of the data, drafting and revision of the manuscript. SP and MD performed the statistical analysis. All authors approved the final manuscript.

## Funding source

The authors would like to thank the Robert Wood Johnson Foundation for their support of this work.

## Pre-publication history

The pre-publication history for this paper can be accessed here:

http://www.biomedcentral.com/1471-2431/12/134/prepub
